# Case Report: Papillon–Lefèvre syndrome beyond keratoderma: two index presentations and sibling screening in two families

**DOI:** 10.3389/fimmu.2026.1888482

**Published:** 2026-07-24

**Authors:** Hilal Karabag Citlak, Fatih Sultan Mehmet Koc, Gulizar Demir, Gulben Ozgul Postuk, Nurdan Kayki Aksoy, Oguzhan Demir, Ayberk Turkyilmaz, Alper Han Cebi, Zeynep Gokce Gayretli Aydin, Gul Salci, Nalan Yildiz, Fazil Orhan

**Affiliations:** 1 Department of Pediatric Immunology and Allergy, Karadeniz Technical University Faculty of Medicine, Trabzon, Türkiye; 2 Department of Pediatric Infectious Diseases, Karadeniz Technical University Faculty of Medicine, Trabzon, Türkiye; 3 Department of Medical Genetics, Karadeniz Technical University Faculty of Medicine, Trabzon, Türkiye; 4 Department of Pediatric Surgery, Karadeniz Technical University Faculty of Medicine, Trabzon, Türkiye

**Keywords:** cathepsin C, diagnostic delay, genotype-phenotype correlation, neutrophil dysfunction, Papillon-Lefèvre syndrome (PLS), primary immunodeficiency diseases, psoas abscess

## Abstract

Papillon–Lefèvre syndrome is a rare inherited disorder classically associated with palmoplantar keratoderma and early severe periodontitis, but its clinical presentation may be broader than expected. We report four molecularly confirmed cases from two unrelated families carrying two different homozygous CTSC variants. The first index patient presented with a large iliopsoas abscess as the initial manifestation of the disease, while her monozygotic twin was diagnosed through family screening and had only mild skin and dental findings. In the second family, a 13-year-old boy had recurrent soft-tissue abscesses and palmoplantar lesions whose diagnosis was delayed for many years by a psoriasis-like presentation; his younger brother was later identified through cascade screening with a skin-limited phenotype. These cases show that PLS may present with deep infections, prolonged diagnostic delay, or mild unrecognized disease. Early molecular testing, careful dental and dermatological assessment, multifocal imaging, and systematic family screening are essential for timely diagnosis and appropriate care.

## 1 Introduction

Papillon–Lefèvre syndrome is characterized by the association of autosomal recessive transgressive palmoplantar keratosis, which begins in early childhood, and rapidly progressive periodontitis during the prepubertal period. The prevalence of this genodermatosis is reported to range from 1 to 4 cases per million, leading to frequent delays in diagnosis in pediatric dentistry (1).

Central to the pathogenesis are loss-of-function mutations in the cathepsin C (*CTSC*) gene in the 11q14.1–q14.3 region (2). This enzymatic deficiency weakens the host defense against periodontal pathogens and is accompanied by loss of function in chemotaxis and phagocytosis steps in neutrophils (3). Mechanistically, cathepsin C (dipeptidyl peptidase I) is required to proteolytically activate the pro-forms of neutrophil serine proteases, namely neutrophil elastase, proteinase 3, and cathepsin G, within maturing neutrophils; in its absence, these effector enzymes remain catalytically inactive, compromising intracellular killing of ingested pathogens (4). Although periodontal and cutaneous disease dominate the classical phenotype, isolated reports have described visceral abscesses, most often hepatic, in patients with PLS, indicating that deep-seated infection is a rare but recognized part of the clinical spectrum (5, 6). The International Union of Immunological Societies (IUIS) classification of inborn errors of immunity lists *CTSC* deficiency (Papillon–Lefèvre syndrome) among the congenital defects of phagocyte number or function within the inborn errors of immunity group (7, 8). Clinically, the onset of keratosis between the first and fourth years of life frequently coincides with the eruption of primary teeth; gingival inflammation, deep periodontal pockets, and prominent fetor develop rapidly, leading to tooth mobility and premature tooth loss.

The existing literature on PLS consists largely of isolated case reports, with limited data on within-family phenotypic variability or on the diagnostic yield of systematic cascade screening following an index diagnosis. Family members sharing the same biallelic *CTSC* variant may differ substantially in disease severity — from life-threatening deep-space infection to subclinical skin changes alone — a variability that complicates clinical recognition and underscores the value of molecular confirmation and structured family evaluation.

On this background, we present four patients with molecularly confirmed PLS from one non-consanguineous (Family 1) and one consanguineous (Family 2) family, harboring distinct homozygous *CTSC* pathogenic variants. These cases collectively illustrate the broad clinical spectrum of the syndrome: an iliopsoas abscess as the inaugural manifestation, a misdiagnosis as psoriasis sustained for thirteen years, and a mild, infection-free phenotype identified exclusively through family screening. Clinical and radiological findings, molecular data, and management approaches are described with the aim of improving early recognition of PLS across its diverse presentations.

## 2 Case presentation

### 2.1 Case 1: index case — iliopsoas abscess as the inaugural presentation

A 2-year-9-month-old female patient presented with a swelling in the right inguinal region that began approximately one month prior. She had used oral amoxicillin-clavulanate prior to presentation, but no significant improvement was observed. On physical examination, she was uneasy but generally stable. A tender, limitedly mobile mass measuring approximately 5 cm was observed on palpation in the right inguinal region. Hyperkeratosis and lamellar peeling, occasionally prominent on the plantar surfaces, were present. Her birth history included a preterm birth from a twin pregnancy at 33 weeks and a two-week neonatal intensive care unit stay due to prematurity. She was fully vaccinated. There was no parental consanguinity, and she had a secundum atrial septal defect under follow-up.

Contrast-enhanced abdominal CT revealed a collection measuring 81×50 mm at its widest point, extending into the right iliac fossa and poorly demarcated from the right iliopsoas muscle in the upper section (**Figure 1**). Both lesions were considered abscesses based on imaging findings. The child underwent surgical drainage, and extended-spectrum intravenous antibiotics were initiated. Abscess cultures grew methicillin-susceptible Staphylococcus aureus (MSSA); an antibiogram revealed resistance to ampicillin, erythromycin, clindamycin, and penicillin, and susceptibility to cefoxitin and trimethoprim-sulfamethoxazole. Treatment was adjusted accordingly. Ultrasonographic follow-up revealed gradual shrinkage of the abscess foci and decreased surrounding inflammation. A small, dense residual collection was observed at final evaluation.

**Figure 1:**
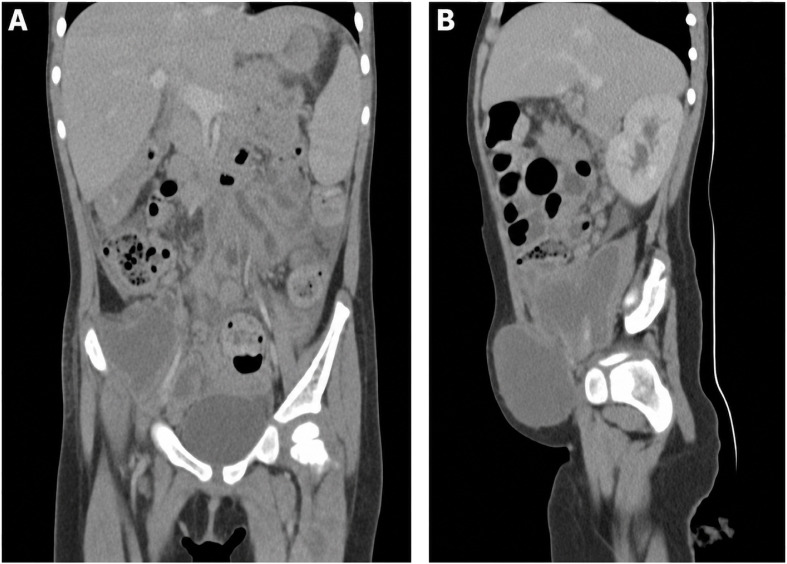
Contrast-enhanced abdominal CT images of Case 1. Coronal **(A)** and sagittal **(B)** reconstructions demonstrate a right iliopsoas/iliac fossa rim-enhancing collection consistent with abscess.

Due to clinical persistence and deep tissue involvement, a persistent focus was investigated. Surgical exploration was performed; necrotic lymph nodes were excised, a tract extending to the psoas was observed, and an appendectomy was performed due to an inflamed appendix. Surgical culture yielded no growth; pathology was reported as consistent with reactive lymphoid hyperplasia and chronic inflammation.

Because the patient presented with a deep-tissue staphylococcal abscess before any genetic result was available, phagocytic disorders — including chronic granulomatous disease (CGD) — were considered alongside primary immunodeficiencies affecting neutrophil function more broadly. A dihydrorhodamine (DHR) flow cytometry oxidative burst assay, which yields a same-day result, was therefore sent in parallel with whole-exome sequencing (WES), the turnaround of which is considerably longer. The DHR assay demonstrated a normal oxidative burst, excluding CGD while the genetic work-up was still pending. WES subsequently revealed the c.815G>C, p.(Arg272Pro) pathogenic variant in the CTSC gene at position 11q14.2, confirming PLS. Monozygosity was supported by parent-of-origin analysis. Sanger sequencing confirmed the same CTSC variant in the patient’s monozygotic co-twin, who presented with overlapping but milder findings and is described separately as Case 2.

Hemodynamic stability was maintained throughout the treatment period, and clinical findings gradually resolved. Infection markers and clinical status were monitored by a multidisciplinary board throughout the hospitalization, and oral hygiene education, moisturizing and keratolytic skin care, and nutritional support were administered. At discharge, the parents were informed about the hereditary characteristics of PLS, the susceptibility to infection, and the importance of early and regular dental follow-up to prevent periodontal disease. Genetic counseling was provided to the family, and extended family screening was recommended. Our patient was followed up in outpatient settings with a discharge plan that included regular checkups, antibiotic treatment completed based on culture results, and warning signs for recurring infections.

### 2.2 Case 2: monozygotic twin of case 1 — mild, infection-free phenotype identified through family screening

The second patient was the monozygotic twin sister of Case 1, born at the same 33-week preterm birth and requiring one week of neonatal intensive care for prematurity. She had no prior history of recurrent infections or deep-tissue abscesses. Following the diagnosis of PLS in her twin sister, she was called in for evaluation, and was first evaluated at the age of 2 years and 11 months.

On physical examination, she had mild bilateral plantar hyperkeratosis and mild hyperhidrosis of the feet. Primary tooth loss beyond what is expected for her age and gingival recession were noted on oral inspection. She had no palpable lymphadenopathy and no signs of active infection at the time of evaluation.

Laboratory evaluation revealed a leukocyte count of 16, 300 cells/µL with an absolute neutrophil count of 9, 850 cells/µL. Hemoglobin was 11.7 g/dL and platelet count was 328, 000/µL. Immunoglobulin levels were within normal limits for age (IgG 970 mg/dL, IgA 96 mg/dL, IgM 78 mg/dL, total IgE 23.5 kU/L). Anti-HBs was reactive at 326.6 IU/L and tetanus antibody was 0.9 IU/mL, both consistent with adequate vaccine responses. Lymphocyte subset analysis showed a CD3 count of 5, 470 cells/µL (62.5%), CD4 of 2, 166 cells/µL (39.6%), CD8 of 981 cells/µL (17.1%), and CD19 of 2, 332 cells/µL (25.4%), all within normal limits. Comprehensive laboratory findings of all cases are presented in **Table 1**.

**Table 1:** Initial laboratory findings.

	Family 1		Family 2	
Parameter	Case 1	Case 2	Case 3	Case 4
Clinical characteristics				
Sex/age at diagnosis	Female, 2y 9m	Female, 2y 11m	Male, 13y	Male, 12y
*CTSC* variant (all homozygous)^a^	c.815G>C (p.Arg272Pro)	c.815G>C (p.Arg272Pro)	c.415G>A (p.G139R)	c.415G>A (p.G139R)
Presenting complaint	Iliopsoas abscess	Mild plantar hyperkeratosis and early primary tooth loss; twin screening	Recurrent abscesses; psoriasis-like rash	Palmoplantar hyperkeratosis; sibling screening
Abscess history	Yes (iliopsoas + inguinal; MSSA)	None	Yes; multiple sites; IV antibiotics required	None
Skin phenotype	Transgressive palmoplantar keratoderma	Mild plantar hyperkeratosis; hyperhidrosis	Erythematous scaly plaques; palms, knees, legs	Palmoplantar hyperkeratosis
Periodontal findings	Age-incompatible findings	Early tooth loss; gingival recession	Not documented at referral	Not documented
Diagnosis route	Index case; WES	Family screening; Sanger	Immunodeficiency gene panel	Family screening; Sanger
Diagnostic delay	—	—	~13 years	~10 years
Haematology				
Hb (g/dL)	8.4 ↓	11.7	15.8	13.9
WBC (cells/µL)	39, 780 ↑	16, 300	7, 360	6, 060
ANC (cells/µL)	36, 230 ↑	9, 850	4, 140	3, 440
ALC (cells/µL)	2, 980	5, 630	2, 320	2, 010
Immunoglobulins				
IgG (mg/dL)	1, 100	970	1, 400	941
IgA (mg/dL)	173	96	168	57
IgM (mg/dL)	152	78	57	50
Total IgE (kU/L)	95	23.5	386.4 ↑	115.9
Vaccine responses				
Anti-HBs (IU/L)	73.5 (reactive)	326.6 (reactive)	2 (non-reactive)	2 (non-reactive)
Tetanus Ab (IU/mL)	0.23	0.9	>5	0.18
Lymphocyte subsets				
CD3% (cells/µL)	66% (5, 227)	62.5% (5, 470)	79% (1, 290)	65.3% (861)
CD4% (cells/µL)	47.2% (3, 768)	39.6% (2, 166)	35.8% (583)	27.3% (417) ↓
CD8% (cells/µL)	NP^b^	17.1% (981)	35.9% (585)	27.4% (419)
CD19% (cells/µL)	11.7% (926)	25.4% (2, 332)	16.9% (275)	14.6% (223)
NK/CD16% (cells/µL)	NP^b^	8.7% (798)	2.7% (47) ↓	NT^c^

WES, whole-exome sequencing; MSSA, methicillin-susceptible *Staphylococcus aureus*; NP, not performed; NT, not tested; ↑, above reference range; ↓, below reference range.

a All four variants confirmed in homozygous state; parental testing confirmed heterozygous carrier status in all cases.

b Reagent unavailable at time of testing.

c Kit unavailable at time of testing.

Haematological indices in Case 1 reflect acute-phase values during active infection. Indices in Cases 2–4 were obtained at elective immunological evaluation.

Sanger sequencing confirmed the *CTSC* c.815G>C (p.Arg272Pro) homozygous variant, establishing the diagnosis of PLS. She was commenced on regular emollient therapy, referred to pediatric dentistry for baseline evaluation and monitoring, and her family was counseled regarding the risk of future infectious complications. Written informed consent was obtained from the parents for inclusion in this report.

### 2.3 Case 3: a decade-long diagnostic delay — a psoriasis-like presentation masking PLS

The third patient was a 13-year-old boy who was referred to the pediatric allergy and immunology outpatient clinic for immunological evaluation of recurrent soft-tissue infections and longstanding skin lesions.

He was born at term. At 25 days of age, he was admitted to the neonatal intensive care unit for delayed umbilical cord separation complicated by omphalitis, alongside hypercalcemia requiring intravenous treatment. Fifteen days after discharge, he was re-admitted for two days with diarrhea and a urinary tract infection. In subsequent years, he experienced recurrent episodes of otitis media, a pyelonephritis resulting in radiological evidence of renal scarring, and multiple soft-tissue abscesses at various sites including the scalp, inguinal, anal, and chin regions. At the age of approximately 18 months, a right cervical abscess necessitated inpatient intravenous antibiotic treatment; a further hospitalization for an abscess was required at around two years of age. Three months prior to the current referral, a right mandibular abscess was managed with oral antibiotics on an outpatient basis.

In parallel with this infectious history, the patient had been followed by dermatology since approximately five years of age for erythematous, scaly plaques on the hands and feet, which his family reported to have been present from birth. The clinical impression at dermatology was palmoplantar psoriasis. A skin biopsy was performed and showed focal parakeratosis with focal accentuation of the granular layer, regular acanthosis, suprapapillar thickening, vertically oriented capillaries in the superficial dermis, and perivascular minimal chronic inflammatory cell infiltration. The pathologist explicitly concluded that the histopathological findings were not consistent with palmoplantar psoriasis. Despite this result, the clinical diagnosis was not revised. The patient was treated sequentially with acitretin 10 mg (self-discontinued by the family due to headache), clobetasol topical corticosteroid for three weeks without benefit, a further course of acitretin, topical corticosteroid in combination, and subsequently calcipotriol/betamethasone dipropionate cream — all with suboptimal and transient responses.

His family history was notable for first-cousin consanguinity between his parents, recurrent furunculosis in his father, and psoriasis-like skin lesions in his younger brother (Case 4). On physical examination at referral, he had erythematous scaly plaques on the palms and knees, and a fluctuant, abscess-like lesion at the right mandibular angle. He was also under follow-up by pediatric endocrinology for a thyroid colloid cyst and nodule, which were considered incidental findings unrelated to the subsequent diagnosis.

Laboratory investigations showed a leukocyte count of 7, 360 cells/µL with an absolute neutrophil count of 4, 140 cells/µL, hemoglobin 15.8 g/dL, and platelet count of 276, 000/µL. Immunoglobulin levels were IgG 1, 400 mg/dL, IgA 168 mg/dL, IgM 57 mg/dL, and total IgE 386.4 kU/L. Tetanus antibody was greater than 5 IU/mL, whereas anti-HBs was 2 IU/L and therefore below the protective threshold. This finding was interpreted cautiously, as an isolated low anti-HBs level does not necessarily indicate a primary antibody production defect. Lymphocyte subset analysis demonstrated a CD3 count of 1, 290 cells/µL (79%), CD4 of 583 cells/µL (35.8%), CD8 of 585 cells/µL (35.9%), and CD19 of 275 cells/µL (16.9%), all within normal limits; however, the CD16+ natural killer cell count was markedly reduced at 47 cells/µL (2.7%).

Given the combination of treatment-refractory palmoplantar skin disease with a biopsy not supporting psoriasis, recurrent deep-tissue infections, and first-cousin parental consanguinity, genetic analysis was requested. Targeted gene panel sequencing (immunodeficiency panel) identified the *CTSC* c.415G>A (p.G139R) homozygous variant, classified as pathogenic by ACMG criteria and previously documented as a disease-causing mutation in ClinVar and HGMD databases. This confirmed the diagnosis of PLS. Parental carrier confirmation and sibling testing, conducted as part of routine diagnostic genetic workup, revealed the variant in heterozygous state in both parents and in homozygous state in his younger brother, who is presented as Case 4. The patient was commenced on emollient therapy, referred to pediatric dentistry, and referred back to dermatology for skin management in light of the revised diagnosis. Psoriasis-directed therapies (retinoids, topical corticosteroids) were discontinued, and management was reoriented toward skin-barrier–focused care, including regular emollient application and keratolytic skin care, with topical corticosteroids reserved for flares. Antibiotic prophylaxis strategies and the importance of early clinical evaluation at fever onset were discussed with the family.

### 2.4 Case 4: younger sibling of case 3 — skin-limited phenotype identified through cascade screening

The fourth patient was the younger brother of Case 3, a 12-year-old boy at the time of first evaluation by pediatric allergy and immunology. He had first been seen by dermatology at the age of four for progressive thickening of the palms and soles that had begun at approximately two years of age; palmoplantar psoriasis was suspected and acitretin was prescribed, but symptoms continued to worsen over subsequent years.

His referral to the allergy and immunology clinic at the age of 11 occurred for a different reason: he had developed a widespread cutaneous rash following consumption of ground meat, and had a prior history of a tick bite. Specific IgE to red meat was 37.8 kU/L and skin prick testing to lamb was positive, supporting a diagnosis of red meat allergy, for which dietary elimination was recommended. In the context of his longstanding palmoplantar skin changes and parental consanguinity, immunological investigations were also performed.

These revealed a leukocyte count of 6, 060 cells/µL with an absolute neutrophil count of 3, 440 cells/µL, hemoglobin 13.9 g/dL, and platelet count of 276, 000/µL. Immunoglobulin levels were IgG 941 mg/dL, IgA 57 mg/dL, IgM 50 mg/dL, and total IgE 115.9 kU/L. Anti-HBs and tetanus antibody titres were both below protective thresholds. Lymphocyte subset analysis obtained at a subsequent visit showed an absolute lymphocyte count of 1, 530 cells/µL, with CD3 at 65.3% (861 cells/µL), CD4 at 27.3% (417 cells/µL, mildly reduced), CD8 at 27.4% (419 cells/µL), and CD19 at 14.6% (223 cells/µL); NK cell enumeration could not be performed due to reagent unavailability.

The diagnosis of PLS was established through cascade family analysis following the index diagnosis in his younger brother: the *CTSC* c.415G>A (p.G139R) variant was found in homozygous state on segregation testing. Notably, this patient had no history of recurrent infections or abscesses at any point. He was commenced on emollient and topical corticosteroid therapy, referred to pediatric dentistry for baseline periodontal evaluation, and enrolled in regular multidisciplinary follow-up. His family was informed about the risk of future infectious complications and advised on early clinical assessment at fever onset. Written informed consent was obtained from the parents for all patients described in this report, and all data were anonymized in accordance with privacy principle.

## 3 Discussion

The microbial basis for visceral and deep tissue abscesses in PLS has been linked to a defect in the maturation of neutrophil serine proteases (neutrophil elastase, proteinase 3, and cathepsin G) via the cathepsin C (dipeptidyl peptidase-1, DPPI) axis; experimental and structural data have shown that DPPI is an essential activator of these proteases (4). Taken together, the four cases presented here illustrate that PLS can manifest across a clinical spectrum far broader than its classical description captures — from a life-threatening retroperitoneal abscess as the inaugural sign of disease, to a decade-long misclassification as psoriasis, to a mild, infection-free palmoplantar phenotype identified only through cascade family screening. Each of these presentations carries distinct diagnostic and management implications, which are explored below.

One of the most clinically consequential findings emerging from this series is the protracted diagnostic delay observed in Cases 3 and 4. In Case 3, palmoplantar skin lesions were present from birth, yet the diagnosis of PLS was not reached until the age of 13 — an interval of approximately thirteen years during which the patient was managed as having psoriasis and exposed to years of retinoid and topical corticosteroid therapy. A skin biopsy obtained during the dermatological workup explicitly concluded that the histopathological findings were not consistent with palmoplantar psoriasis; despite this objective finding, a diagnostic reassessment was not pursued — a pattern that reflects the broader challenge of distinguishing palmoplantar keratoderma from psoriasis in the absence of prominent periodontal findings or a molecular diagnosis that would prompt genetic evaluation. The palmoplantar keratoderma of PLS shares several features with psoriasis on clinical inspection — erythema, scaling, and hyperkeratosis — and distinguishing between them on morphology alone can be challenging, particularly when dental findings are not yet prominent or are not sought. However, when a biopsy fails to confirm psoriasis in a child with palmoplantar skin disease, especially in the context of consanguinity or a history of recurrent infections, PLS should be actively reconsidered. Periodic acid-Schiff staining and histopathological review in concert with clinical data may aid this distinction, but molecular confirmation remains the definitive step. In Case 4, who is the younger sibling of Case 3, the diagnosis was similarly delayed by approximately ten years; in his case, the correct diagnosis was ultimately reached not through clinical re-evaluation but through cascade genetic testing following his brother’s diagnosis — underscoring how dependent timely detection can be on whether an index case is identified in the family.

The relative rarity of fulminant infection in PLS, compared with the prominent oral and cutaneous involvement, remains incompletely understood. Murine models lacking DPPI show a broader immunodeficiency phenotype than humans: loss of serine-protease activation predisposes these mice to staphylococcal, E. coli, and fungal infections, and mice deficient in granzymes A and B show impaired cytotoxic-lymphocyte–mediated killing and defective antiviral responses (9). By contrast, humans with CTSC deficiency do not display a comparable T-cell– or NK-cell–mediated immunodeficiency; residual granzyme activity and largely preserved cytotoxic lymphocyte function persist despite loss of neutrophil serine-protease maturation, and cathepsin C–independent pathways of granzyme B activation have been demonstrated in interleukin-2–activated human NK cells (10). This species-specific divergence may help explain why most patients with PLS, despite a shared enzymatic defect, present with localized periodontal and cutaneous disease rather than a generalized susceptibility to severe infection, and why abscess formation — as seen in our index case — remains an uncommon rather than defining feature.

Cases 1 and 2 harbor the *CTSC* c.815G>C (p.Arg272Pro) variant in homozygous state, while Cases 3 and 4 carry the c.415G>A (p.G139R) variant. Both variants have been classified as pathogenic; Arg272Pro was first reported in homozygous state by Toomes et al. (11) in a patient with palmoplantar keratosis and periodontitis, and subsequently identified by Pham et al. (9) in compound heterozygous state alongside a truncating variant in a patient with recurrent staphylococcal infections including a subhepatic and a cerebral abscess. The Arg272Pro substitution falls within the second turn of the central helix from the carboxy terminus of cathepsin C, within the heavy chain, and is predicted to distort the three-dimensional structure and abrogate enzyme activity (12). The p.G139R substitution, identified in Cases 3 and 4, replaces glycine — the smallest and conformationally most flexible amino acid — with the bulkier, positively charged arginine; this substitution is similarly expected to impair correct protein folding and has been previously classified as a disease-causing mutation in both ClinVar and HGMD databases. Neither variant permits a strong genotype-phenotype correlation on its own. This series reinforces that observation: Cases 1 and 2, who share the same Arg272Pro variant within the same family, differ markedly in clinical severity — one presented with a life-threatening iliopsoas abscess, while her monozygotic twin had only mild cutaneous and dental findings at the same age. An analogous pattern is seen in Family 2, where Case 3 accumulated multiple deep-tissue infections over thirteen years while Case 4, carrying the identical G139R variant, has remained free of infectious complications. The within-family phenotypic variability observed across both families suggests that modifier genes, environmental exposures, or stochastic factors in neutrophil function likely contribute to determining which patients develop severe infectious disease, and argues against attributing infection risk to the specific variant alone. The clinical photographs further illustrate this phenotypic variability, ranging from mild plantar involvement to marked bilateral palmar hyperkeratosis and early primary tooth loss (**Figure 2**).

**Figure 2:**
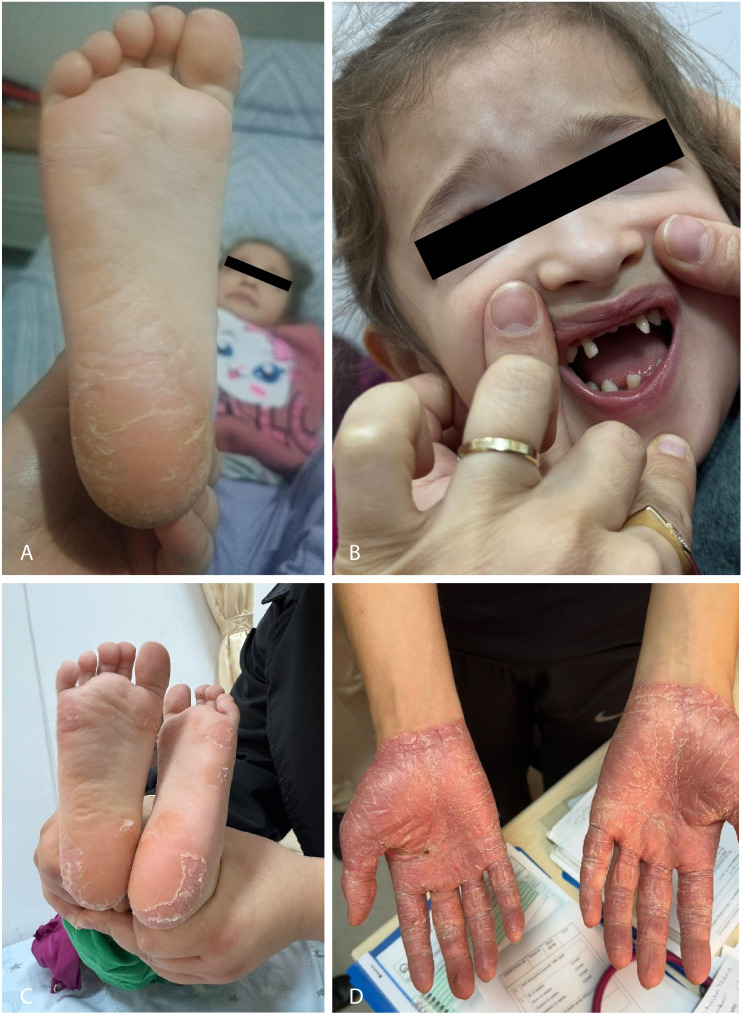
Clinical manifestations of Papillon–Lefèvre syndrome across two unrelated families. **(A)** Plantar hyperkeratosis in the Family 1 index case, who presented with an iliopsoas abscess. **(B)** Early primary tooth loss in the monozygotic twin of the Family 1 index case, identified through family screening. **(C)** Bilateral plantar hyperkeratosis in the same twin sibling. **(D)** Bilateral palmar hyperkeratosis in the Family 2 index case, who had a prolonged psoriasis-like presentation before molecular diagnosis.

The liver is the most common visceral site in the literature for PLS-associated abscesses; accumulated case reviews and pediatric series highlight liver abscesses as an increasingly recognized complication of PLS, frequently reporting Staphylococcus aureus as the causative agent (5). In this context, systematically including the hepatic focus in early imaging algorithms is recommended, and hematogenous spread from periodontal disease has been implicated in the pathogenesis. However, the index case in this series — presenting with an 81×50 mm iliopsoas abscess and a concomitant inguinal collection as the inaugural and clinically dominant presenting feature of PLS — extends the recognized anatomical spectrum of PLS-associated infections to the musculoretroperitoneal compartment. Cerebral and renal parenchymal abscesses have been reported in PLS, albeit rarely, and even renal cases can mimic malignancy on imaging (6). The present series therefore reinforces the need for a multifocal scanning strategy that encompasses hepatic, neuraxial, urinary, retroperitoneal, and muscular regions in any child with PLS presenting with fever or an unexplained inflammatory response, rather than an imaging approach anchored exclusively to the hepatic focus.

The value of systematic cascade family screening is demonstrated by both families in this series. In Family 1, evaluation of the monozygotic twin following the index diagnosis identified a child with early dental and cutaneous features of PLS who had not yet developed infectious complications; early recognition now allows for anticipatory guidance, dental monitoring, and parental education regarding infection risk. In Family 2, the index diagnosis in Case 3 led to identification of his younger brother, who had been followed for years with an incorrect diagnosis of psoriasis. These observations suggest that in any confirmed case of PLS, evaluation of all first-degree relatives — including siblings who may carry a similar unrecognized phenotype — should be regarded as a standard component of management rather than an optional step.

Selected immunological findings across the four cases merit brief comment. Low or non-protective vaccine antibody titres were observed in Cases 3 and 4; however, because PLS is not primarily a disorder of antibody production, these isolated serological findings should be interpreted cautiously and require reassessment after booster vaccination if clinically indicated. In Case 3, the CD16+ natural killer cell count was markedly reduced at 47 cells/µL (2.7% of lymphocytes); whether this reflects a direct immunological consequence of *CTSC* deficiency or a secondary effect of chronic infectious burden remains to be determined with longitudinal follow-up. In Case 4, a mild reduction in absolute CD4 count was observed (417 cells/µL), a finding worth noting in the context of future immunological characterization of PLS. Total IgE was mildly to moderately elevated in Case 3 (386.4 kU/L). However, this level was not in the range typically associated with hyper-IgE syndromes, and it was not interpreted as a defining immunological feature of PLS. Rather, it was considered a nonspecific finding in the context of chronic skin inflammation and recurrent infections.

Two cases of Papillon–Lefèvre syndrome have shown that short-term systemic corticosteroid supplementation in liver abscesses may contribute to drainage and antimicrobial response by reducing the thick inflammatory sheath; however, the level of evidence is case report level, this approach is limited to selected hepatic foci, and cannot be generalized to psoas and deep-compartment abscesses (13).

In the index case, the isolation of methicillin-susceptible Staphylococcus aureus from the abscess culture supports the predominance of S. aureus in PLS-associated infections. In patients prone to recurrence, it is prudent to consider carrier screening and decolonization protocols in selected cases, alongside family hygiene measures (14). While there are no specific guidelines for PLS, the Infectious Diseases Society of America (IDSA) guidelines support this approach, conditionally recommending decolonization for recurrent S. aureus infections (15).

### 3.1 Limitations

This report has several limitations. First, as a case series of four patients from two families, it does not permit epidemiological conclusions about the true frequency of atypical presentations within the broader PLS population. Second, the diagnostic and therapeutic decisions described here were made in the course of routine clinical care rather than within a predefined research protocol, and no additional experimental or mechanistic data were generated to explain the observed phenotypic heterogeneity. Third, longitudinal outcome data, particularly regarding treatment response in Case 3 and the natural course of the reduced NK-cell count observed in that patient, remain limited; continued follow-up will be needed to clarify these findings. Finally, because Cases 2 and 4 were identified through family screening rather than independent clinical presentation, this series may underrepresent the true range of subclinical PLS phenotypes that exist in the wider population.

In summary, this case series demonstrates that PLS can present in ways that diverge substantially from its classical description. An iliopsoas abscess as the first manifestation, a psoriasis-like phenotype persisting for more than a decade, and disease detected only through family screening are all part of the same diagnostic spectrum. In children with recurrent soft-tissue infections or treatment-refractory palmoplantar skin disease — particularly in the setting of consanguinity or a family history of similar findings — PLS should be considered and molecular confirmation sought without delay. Systematic multifocal imaging not limited to the hepatic focus, timely and culture-guided source control, simultaneous reduction of periodontal microbial load, and proactive family screening form the cornerstones of a comprehensive management approach in these patients.

## Data Availability

The de-identified data supporting the conclusions of this article are available from the corresponding author upon reasonable request, subject to applicable ethical and patient privacy restrictions. No new publicly accessible genomic dataset was generated or deposited as part of this case report.
